# Pimozide Inhibits CatSper Activity, Impairs Hyperactivation and the Acrosome Reaction in Human Spermatozoa

**DOI:** 10.3390/ijms27125357

**Published:** 2026-06-13

**Authors:** Jorge Arturo Torres Juárez, Ana Gabriela Hernández Puga, Esperanza Mata Martínez, Claudia Lydia Treviño Santa Cruz, Ana Alicia Sánchez Tusie

**Affiliations:** 1 Centro de Investigación Biomédica Avanzada, Facultad de Medicina, Universidad Autónoma de Querétaro (UAQ), Santiago de Querétaro 76010, Mexico; jtorres65@alumnos.uaq.mx (J.A.T.J.); ana.gabriela.hernandez@uaq.mx (A.G.H.P.); 2 Departamento de Biología Celular y Desarrollo, Instituto de Fisiología Celular, Universidad Nacional Autónoma de México (UNAM), Ciudad de México 04510, Mexico; espemmtz@gmail.com; 3 Departamento de Genética del Desarrollo y Fisiología Molecular, Instituto de Biotecnología (IBT), Universidad Nacional Autónoma de México (UNAM), Cuernavaca 62210, Mexico; claudia.trevino@ibt.unam.mx

**Keywords:** male contraception, CatSper, molecular dynamics, pimozide

## Abstract

Health, social, and ethical considerations highlight the need for new male contraceptives. Pimozide is an FDA approved drug known to block T-type calcium channels and which shares structural similarities with mibefradil, a proven antagonist of the CatSper channel. In this study, we examined the effect of pimozide on CatSper, a key target for non-hormonal male contraception. Molecular docking and molecular dynamics simulations were carried out to assess how pimozide binds within the channel pore, and binding energies were estimated using MM-GBSA. To determine its impact on sperm function, we evaluated hyperactivation, the acrosome reaction, and CatSper activity. Our computational analyses indicate that pimozide functions as a pore blocker of the CatSper channel. Experimental findings further support this, showing that pimozide inhibits CatSper activity, and impairs hyperactivation and the acrosome reaction in human spermatozoa. Overall, these results identify pimozide as a novel CatSper antagonist and propose a binding mode, offering a basis for the rational design of reversible, non-hormonal male contraceptives that target the CatSper channel.

## 1. Introduction

The need for novel male contraceptives arises from several concerns: the rapid growth of the global population and its environmental impact [1], the high incidence of unintended pregnancies and their negative consequences for health and socioeconomic stability [2], the limited options and drawbacks of current male contraceptives [3], and the ethical imperative to share responsibility in family planning [4].

Novel male contraceptive methods are generally classified into hormonal and non-hormonal approaches [5]. Hormonal methods have shown promise in clinical studies, but side effects have hindered their progress toward commercialization [6]. In contrast, non-hormonal strategies are considered more attractive. Mechanical approaches, such as vas deferens blockers, have advanced to clinical trials [7], and one target-based strategy is also in testing [8]. However, the high attrition rates in drug development highlight the importance of pursuing alternative molecular targets.

The CatSper channel, a sperm-specific cation channel essential for male fertility, is an attractive non-hormonal target [9,10]. CatSper is a complex ion channel composed of at least 14 subunits, four α-subunits form its heterotetrameric pore region. CatSper plays a vital role during sperm capacitation, being the principal channel responsible for calcium influx in spermatozoa. This influx of calcium is needed for sperm to acquire hyperactivation, an asymmetric motile pattern that allows spermatozoa to navigate the viscous female reproductive tract. For CatSper, several antagonists have been reported [11,12,13,14,15,16,17,18,19,20], but structural insights remain limited, slowing drug development. To accelerate this process, drug and scaffold repurposing are valuable strategies to consider.

Here, we investigated the potential of pimozide, an antipsychotic drug approved for tics treatment in Tourette’s syndrome [21] as an antagonist of CatSper. Particularly, we choose pimozide for a variety of reasons. Since pimozide is an FDA approved drug, repurposing holds the potential to hasten the development of male contraceptives. Furthermore, pimozide is structurally similar to mibefradil, a known antagonist of CatSper. Finally, since it’s known that pimozide acts as a pore blocker to other calcium channels, we could evaluate in silico the pore blocking action of pimozide on the CatSper channel.

## 2. Results

### 2.1. Homology Modelling

For the determination of the three-dimensional structure of the CatSper, we employed homology modelling. For this, we used the α-subunits of the known structure of the mouse Catsper channel, which is in a presumed closed conformation due to its pore dimensions. The amino acid sequence of α-subunits of the human CatSper channel has mean 66.23% identity with the mouse CatSper channel. Since this value exceeds the minimum rule of thumb of 30%, we used the SWISS-MODEL server for the generation of the model. With respect to its structural quality, over 95% of the amino acids of the model are in favorable regions of the Ramachandran plot (Figure S1a). Furthermore, the model presents a score of 0.85 in QMEANbrane (https://swissmodel.expasy.org/qmean/, accessed on 1 January 2026), a web software specialized in quality assessment of membrane proteins (Figure S1b). Taking these results together, our model presents optimal stereochemistry and local geometry.

### 2.2. Molecular Docking and Molecular Dynamics

After evaluating the quality of our model, we proceeded with the docking of pimozide. Since the pore of the human Cav3.3 channel is the binding site of pimozide and mibefradil, a known antagonist of CatSper, we selected the pore of CatSper channel as the binding site. The docking score of the top pose was −10.9 kcal/mol, indicating a favorable interaction.

**Figure 1:**
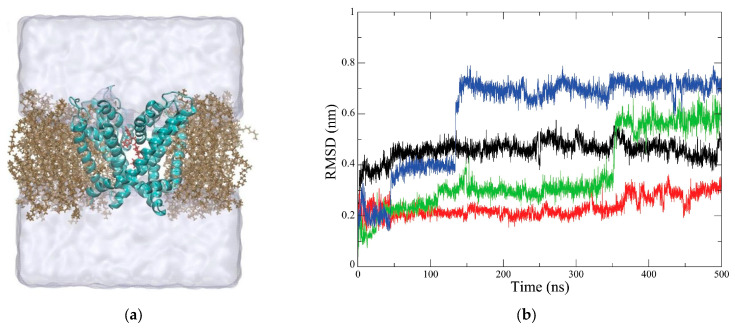
Molecular dynamics of the CatSper-Pimozide complex. (**a**) Representation of the molecular system. The pore of the CatSper channel is shown in cyan, embedded in a POPC bilipid layer in brown. Pimozide is shown in red, occluding the pore. (**b**) RMSD of the complex during 500 ns of simulation. Each color represents a replica of the system.

To evaluate the stability of pimozide on the pore of the CatSper channel, we simulated the system for 500 ns (Figure 1a). We saw that pimozide remained in the pore of the channel throughout the simulation time period (Video S1), and that after 350 ns, all the four replicas remained stable, with two of the four replicas presenting two or more transient stable conformations (Figure 1b).

**Figure 2:**
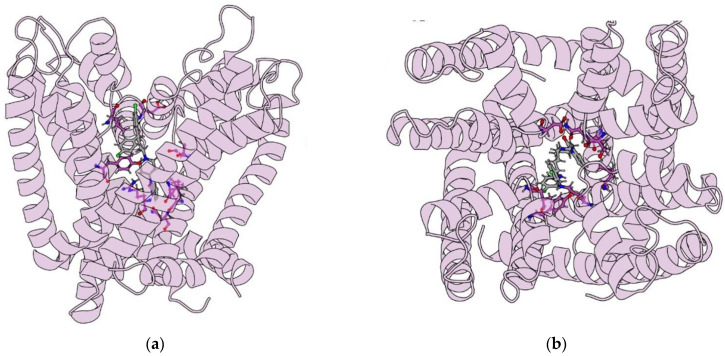

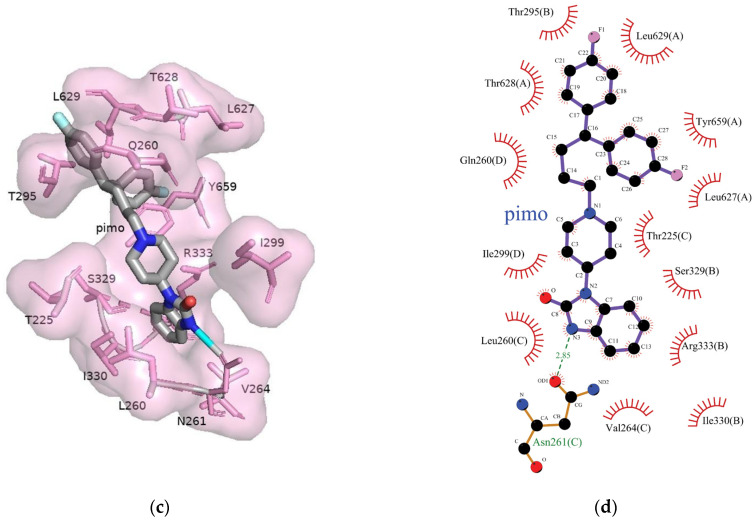
Binding mode of the most representative cluster after 400 ns. (**a**,**b**) pimozide-CatSper side and top-down view. (**c**,**d**) binding mode of pimozide within the pore of CatSper. Pimozide interacts with CatSper mainly through hydrophobic interaction with its ring system.

To obtain the most representative binding mode, we selected the top cluster of the last 100 ns of each simulation. Hydrophobic interactions between the rings of pimozide and the subunits of CatSper appeared consistently in all replicas (Figure 2). Hydrophobic interactions are mediated by the fluorophenyl rings and the benzimidazole ring. Hydrogen bonds were formed in the piperidine ring in its amide group in three of the four replicas with an asparagine, and in the fluorine atoms in two of the four replicas (Figure S2).

Finally, to evaluate the binding affinity of pimozide, we estimated the Gibbs free energy with MM-GBSA. Here, we saw that pimozide presents a favorable binding energy (−109.856 kcal/mol, Table S1), which prompts us to evaluate its effect on human spermatozoa.

### 2.3. Sperm Hyperactivation

**Figure 3:**
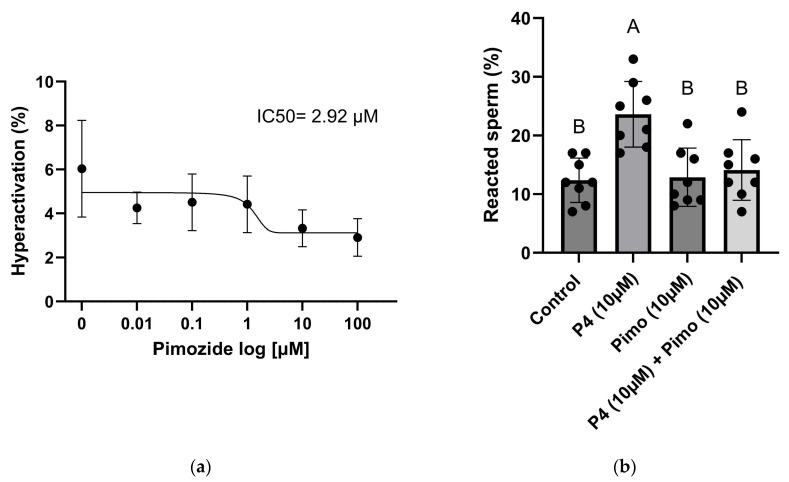
Pimozide alters the functionality of human spermatozoa. (**a**) Human sperm were exposed to pimozide (0–100 µM) incubated for 2 h. Hyperactivation was evaluated in at least 5 different fields with OpenCASA. (**b**) After 4 h of capacitation with or without pimozide 10 µM (Pimo), spermatozoa were exposed to progesterone (P4) or DMSO (control). Results are expressed as mean ± SEM. Different letters indicate statistical differences, n = 8.

The hallmark of CatSper function is sperm hyperactivation [12]. During capacitation, sperm exhibit hyperactivated motility, a pattern dependent on calcium entry via CatSper, which facilitates movement through the viscous female reproductive tract [22]. Due to this, we asked if exposing sperm to pimozide could inhibit the acquisition of hyperactivation. For this, we choose concentrations of pimozide (0, 0.01, 0.1, 1, 10 and 100 µM), overlapping the peak serum concentration of 0.04 µM [23]. Here, we saw a reduction in hyperactivated spermatozoa exposed to increasing concentrations of pimozide. Pimozide acted in a dose-response manner on hyperactivation with an IC50 of 2.92 µM (Figure 3a).

### 2.4. Acrosome Reaction

Another key function of sperm for human fertilization is the acrosome reaction (AR). This reaction is an exocytotic process that depends on calcium and allows sperm to bind to the ovum [24]. Though the involvement of CatSper in the AR is debated [9,25], this reaction can be induced with progesterone, an agonist of CatSper and a physiological inductor of the AR [15,16,24]. To compare the potency of pimozide with other antagonists of CatSper, we evaluated its effect using a concentration of 10 µM pimozide. Here, we saw that the number of reacted spermatozoa was significantly higher in the group exposed to progesterone than in the control group. In contrast, spermatozoa exposed to pimozide maintained the same levels of reacted sperm as the control group, even in the presence of progesterone (Figure 3b).

### 2.5. CatSper Activity

**Figure 4:**
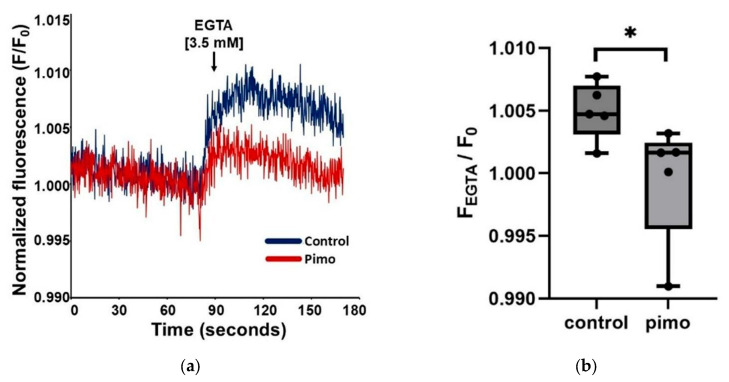
Pimozide inhibits the activity of CatSper in non-capacitating conditions. After 5 min of basal DISC3(5) fluorescence recording, 3.5 mM of EGTA was administered to the cuvette. (**a**) Representative traces of the CatSper activity assay in non-capacitated sperm. (**b**) CatSper activity in the presence or absence of 10 µM pimozide, in non-capacitated sperm, n = 5. * *p* < 0.05.

While the detrimental effect of pimozide on functions dependent on calcium entry to sperm is suggestive of its action on CatSper, there is a need to employ validated assays of the activity of CatSper [26]. For this, we measured with fluorimetry the changes in the membrane potential of a population of spermatozoa after calcium chelation in the presence or absence of pimozide. In the absence of extracellular calcium, an influx of sodium mediated by CatSper depolarizes the sperm membrane. We saw that sperm exposed to pimozide in non-capacitated conditions respond significantly less to the chelation stimulus (Figure 4b).

## 3. Discussion

Despite the growing demand for male contraceptives, no new options have been approved. To provide a framework for the characterization of novel antagonists of CatSper, we examined with computational and experimental approaches the potential of pimozide as a pore blocker of the CatSper channel.

Molecular docking and dynamics simulations revealed favorable interactions between pimozide and the CatSper pore. The docking scores, stability of the complex, and binding free energy rescoring with MM-GBSA indicate strong binding. The predicted binding mode involves hydrophobic interactions and hydrogen bonds. This aligns with the known binding of pimozide to the human Cav3.3 channel at the fenestration site between the selectivity filter and the intracellular gate and shares the formation of a hydrogen bond between an asparagine and the benzimidazole group [27]. Interestingly, while the fluorophenyl groups of pimozide in Cav3.3 are oriented toward the intracellular gate, our model positions them near the selectivity filter, suggesting potential avenues for selectivity optimization. This is worth exploring considering the IC50 of pimozide on Cav3.3 vs. CatSper (0.77 vs. 2.92 µM) [27]. Enhancing shape complementarity between ligand and receptor, for example, by introducing hydrophobic moieties to occupy the binding cavity, is a proven strategy to improve affinity and reduce off-target effects [28]. Indeed, structure-activity relationship studies have applied this approach to pimozide, successfully reducing its affinity for dopamine receptors [29], reinforcing its potential as a scaffold for male contraceptive development. However, future studies, using patch-clamp electrophysiology and experimental structure determination techniques, are needed to validate the pore blocking action of pimozide and the proposed binding mode.

To explore our computational findings, we assessed the effects of pimozide on human sperm function. Pimozide induced a dose-dependent decrease in the percentage of hyperactivated sperm, consistent with previous reports on other CatSper antagonists, including RU1968 [18], mibefradil, ML218, medroxyprogesterone acetate, levonorgestrel, aldosterone [11], HC-056456 [12], and NNC55-0396 [30]. With respect to the IC50 of pimozide, this was comparable to the IC50 of other antagonists of CatSper (Table 1).

The AR also depends on calcium entry [24]. Pimozide has been previously shown to reduce the AR induced with solubilized zona pellucida in human sperm [31]. Here, we looked at the effect of pimozide in the AR induced with progesterone and observed similar results. Since the zona pellucida elicits the AR by stimulating CatSper via a different mechanism than progesterone [32], our results argue in favor of a direct action of pimozide on CatSper.

Other antagonists of CatSper reported to reduce the percentage of AR induced by progesterone are: RU1968 [18], HC-056456 [12], mibefradil [25], and sertraline [19]. Similarly to others [18], pimozide did not increase the spontaneous AR (sAR) percentage, sAR is the acrosome reaction that occurs without the presence of any inducer, like progesterone that can be used as a positive control. However, some studies have reported an increase in the sAR in the presence of known inhibitors in capacitated [20,33] and non-capacitated sperm [34].

To evaluate the activity of CatSper in a validated assay, we measured the changes in the membrane potential before and after chelating extracellular calcium in the presence or absence of pimozide. We saw that pimozide was capable of reducing the response of this chelating stimulus. Future studies could explore ligand binding to the channel in an open state and assess different incubation times. Moreover, our study was limited by not modeling additional CatSper subunits, restricting the evaluation of other potential binding sites. Finally, due to the reported cardiotoxicity of pimozide and its known ability to block T-type calcium channels and dopamine receptors, development of male contraceptives based on this scaffold must carefully consider selectivity profiles or possible side effects.

## 4. Materials and Methods

### 4.1. Homology Modeling

The structure of the human CatSper channel was modeled using the SWISS-MODEL server (https://swissmodel.expasy.org, accessed on 1 January 2026) [35]. The α-subunits from the Catspermasome structure (RCSB 7EEB) served as templates [36], and structural alignment was performed to reconstruct the heterotetrameric, pore-forming region of the channel. The model was protonated at pH 7.4 using the AddH module in Chimera v1.15 [37]. Model quality was assessed via Ramachandran plots, while local geometry was evaluated using the QMEANbrane web server [38].

### 4.2. Molecular Docking

The structure of pimozide was recovered from the Drug Bank database [39] and was energy-minimized in Avogadro V1.2.0 with the MMFF94 force field and the steepest descent algorithm [40]. Open Babel V3.1.1 was used to convert to the PDBQT format [41]. Next, the CatSper pore was selected as the docking site. A grid box of 26 × 26 × 26 Å covering the pore was used, and docking was conducted with AutoDock Vina (version 1.2.7) [42]. The highest-scoring pose of the pimozide-CatSper complex was selected for molecular dynamics simulations.

### 4.3. Molecular Dynamics

The complex was embedded in a POPC membrane, solvated with TIP3P water, and neutralized with Na^+^ and Cl^−^ ions at 0.15 M using CHARMM-GUI (https://www.charmm-gui.org, accessed on 1 January 2026) [43]. Simulations were run in GROMACS 2023.5 [44] with the CHARMM36f force field [45], and pimozide topology was assigned via CGenFF [46]. Electrostatics were treated with the Particle Mesh Ewald method, and all bonds were constrained with LINCS. Energy minimization was performed using the steepest descent algorithm for 5000 steps or until the maximum force fell below 1000 kJ·mol^−1^·nm^−1^. The system was equilibrated according to the six-step CHARMM-GUI protocol, followed by four 500 ns production simulations in the NPT ensemble at 310.15 K and 1 atm using the v-rescale thermostat and Parrinello-Rahman barostat.

### 4.4. Trajectory Analysis and Binding Free Energy

Root mean square deviation (RMSD) was calculated with GROMACS tools. The relative binding free energy was estimated using gmx_MMGBPBSA (version 1.6.4) [47], analyzing all frames from the last 100 ns of each replica at 310.15 K and 0.15 M ionic strength with the GBneck (igb7) model. The mean structure of the top cluster from the last 100 ns was selected to represent a plausible binding mode. Molecular interactions were analyzed using Ligplot+ v2.2.9, PyMOL v3.1.6.1 and the Protein Imager server (https://3dproteinimaging.com/protein-imager, accessed on 1 January 2026) [48,49,50].

### 4.5. Semen Sample Collection

For the in vitro portion of the study, ethical approval from the Ethical Committee of the Faculty of Medicine, Autonomous University of Queretaro (protocol code BIOM-013/2024-6 and date of approval 28 June 2024) was obtained. Next, 8 male participants with at least 2 days of sexual abstinence and normal semen parameters according to the WHO manual 5th edition were recruited, excluding participants with known sexually transmitted infections, chronic diseases, use of illicit substances or under pharmacological treatment. After written informed consent, semen samples were requested. According to WHO guidelines, a normal semen analysis typically shows a volume of at least 1.5 milliliters, a sperm concentration of 15 million sperm per milliliter or more, at least 40% motility, and a morphology with at least 4% of sperm having a normal shape. For this, human sperm concentration was measured with a Makler chamber. Semen volume was determined with a serological pipette. Morphology was analyzed with the Diff-Quik stain following the WHO 5th edition strict criteria. Motility was measured by placing 10 µL of the semen sample in a slide with a coverslip of 22 mm by 22 mm, categorizing sperm movement in progressive motility, non-progressive motility and immotility.

### 4.6. Sperm Hyperactivation

Semen samples were liquefied and processed using the swim-up method. Aliquots of 500 µL semen were layered with 500 µL Ham’s F-10 medium (ThermoFisher Gibco catalog number: 11550043, Waltham, MA, USA) in Falcon tubes, inclined at 45°, and incubated at 37 °C with 5% CO_2_ for 1 h. The upper fraction was collected and adjusted to 10 × 10^6^ sperm/mL. Samples were split into six groups exposed to varying concentrations of pimozide (0–100 µM) in a medium containing 5 mg/mL bovine serum albumin for 3 h. Motility was recorded in five fields per sample at 10× magnification (60 fps, 2048 × 1536 pixels), and hyperactivation was determined with OpenCASA based on a lateral head displacement (ALH) ≥ 3.5 µm, mean curvilinear velocity (VLC) > 150 µm/s, and a linearity (LIN) < 50%.

### 4.7. Acrosome Reaction

After swim-up, sperm were capacitated for 4 h with or without 10 µM pimozide, then treated with 10 µM progesterone or 0.1% DMSO for 30 min. Samples were centrifuged, resuspended in cold methanol, smeared, air-dried, and stained with FITC-PSA. At least 100 spermatozoa per sample were analyzed under fluorescence microscopy (498/517 nm).

### 4.8. CatSper Activity

Membrane potential changes in response to EGTA (F_EGTA_) were measured in sperm adjusted to 5–10 × 10^6^/mL and pretreated with 10 µM pimozide before DISC3(5) loading to a final concentration of 1 µM. 3.5 mM of EGTA was added after 5 min of basal fluorescence (F_0_), with fluorescence being recorded at 620/670 nm at 37 °C. F_EGTA/F0_ ratios were used to quantify CatSper activity [26].

### 4.9. Statistical Analysis

The distribution of the variables was evaluated with the Shapiro-Wilk test. Group comparisons were performed using paired Student’s *t*-test for the CatSper Activity dataset, and repeated measures ANOVA followed by Tukey HSD test for the reacted sperm dataset. Three parameter Hill equation was fitted to estimate the half maximal inhibitory concentration (IC50). Data are presented as mean ± standard error, with *p* < 0.05 set for statistical differences. GraphPad Prism 10.2.0 software was used for all statistical analysis.

## 5. Conclusions

Overall, we have characterized in silico and in vitro, the non-hormonal contraceptive potential of pimozide. Our results suggest that pimozide can potentially function as a scaffold for the generation of male contraceptives targeting the complex CatSper channel, though repurposing pimozide as a CatSper antagonist is contingent on in vivo data.

## Figures and Tables

**Table 1:** IC50 comparisons between known CatSper antagonists and pimozide.

Compound	IC50 (µM)	Method of Determination	Reference
RU1968F1	0.4	Patch-clamp	[18]
Tetrahydrocannabinol	1.88	Fluorimetry	[20]
Cannabidiol	2.47	Fluorimetry	[20]
Pimozide	2.92	Hyperactivation	This study
HC-056456	3	Fluorimetry	[12]
RU1968F1	3.1	Fluorimetry	[18]
ML218	5	Fluorimetry	[11]
Medroxiprogesterone	6.1	Fluorimetry	[11]
Sertraline	4.61–16.19	Fluorimetry	[19]
Mibefradil	6.18–18	Fluorimetry	[16]

## Data Availability

The raw data supporting the conclusions of this article will be made available by the authors upon request.

## Supplementary Materials

The following supporting information can be downloaded at: https://www.mdpi.com/article/10.3390/ijms27125357/s1.

## Abbreviations

The following abbreviations are used in this manuscript:

RMSD	Root mean square deviation
EGTA	Ethylene glycol tetraacetic acid
DMSO	dimethyl sulfoxide
MM	Molecular mechanics
GBSA	General Born Surface Area
RCSB PDB	Research Collaboratory for Structural Bioinformatics Protein Data Bank
POPC	1-palmitoyl-2-oleoyl-sn-glycero-3-phosphocholine
FITC-PSA	Fluorescein isothiocyanate-labeled *Pisum sativum agglutinin*
