# Supplementary material for: Pimozide Inhibits CatSper Activity, Impairs Hyperactivation and the Acrosome Reaction in Human Spermatozoa
Source: Int J Mol Sci. 2026 Jun 13;27(12):5357. doi: 10.3390/ijms27125357 (PMC13299526; doi:10.3390/ijms27125357)
Supplement: Supplementary file 1 [file ijms-27-05357-s001.zip › ijms-4039993-supplementary.pdf]

Supplementary information of the article:

Pimozide inhibits CatSper activity, impairs hyperactivation and the acrosome reaction in human spermatozoa

Jorge Arturo Torres Juárez[1], Ana Gabriela Hernández Puga[2], Esperanza Mata Martínez[2], Claudia Lydia Treviño Santa Cruz[2] and Ana Alicia Sánchez Tusie[1,*]

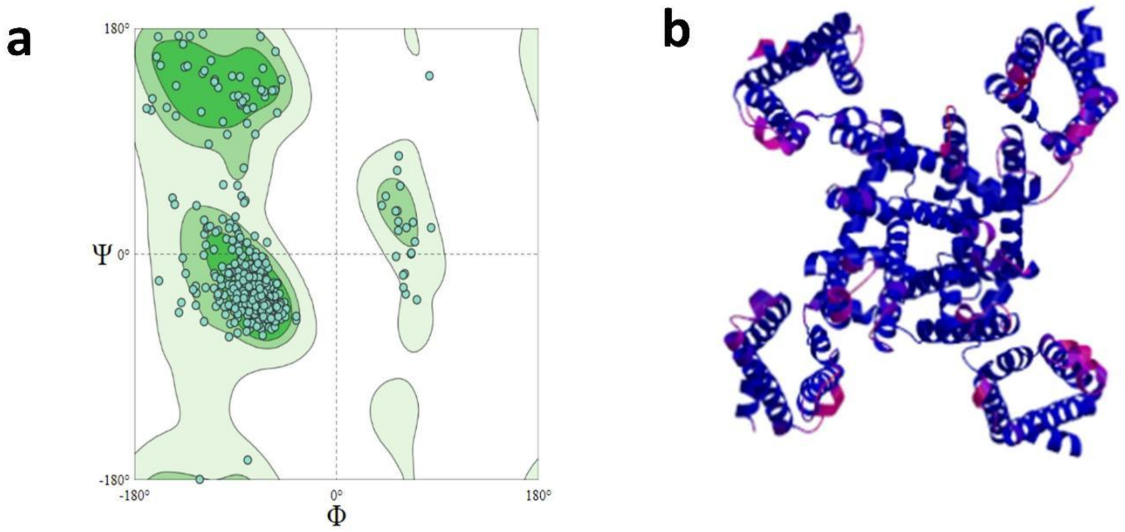

Figure S1. Structural evaluation of the human CatSper channel model. a) Ramachandran plot shows that 95.63% of amino acids are located in the favorable regions of the plot. Similarly, b) QMEANbrane demonstrates a high local quality (score of 0.85).

Video S1 https://youtu.be/CchYev_k59o

.

Table S1. Gibbs free energy components of the complex of CatSper-Pimozide of the last 100 ns of the simulations.

	VDWAALS	EEL	EGB	ESURF	GGAS	GSOLV	TOTAL
1st replica	-78.28	-171.65	150.62	-11.1	-251	139.52	-111.47
2nd replica	-66.56	-233.12	214.58	-10	-301.21	204.58	-96.63
3rd replica	-72.26	-159.83	136.96	-10.11	-233.12	126.85	-106.27
4th replica	-71.77	-192.31	158.86	-10.15	-265.23	148.71	-116.52
mean	-73.768	-187.398	162.886	-10.506	-262.238	152.38	-109.856

All values are expressed in kcal/mol.

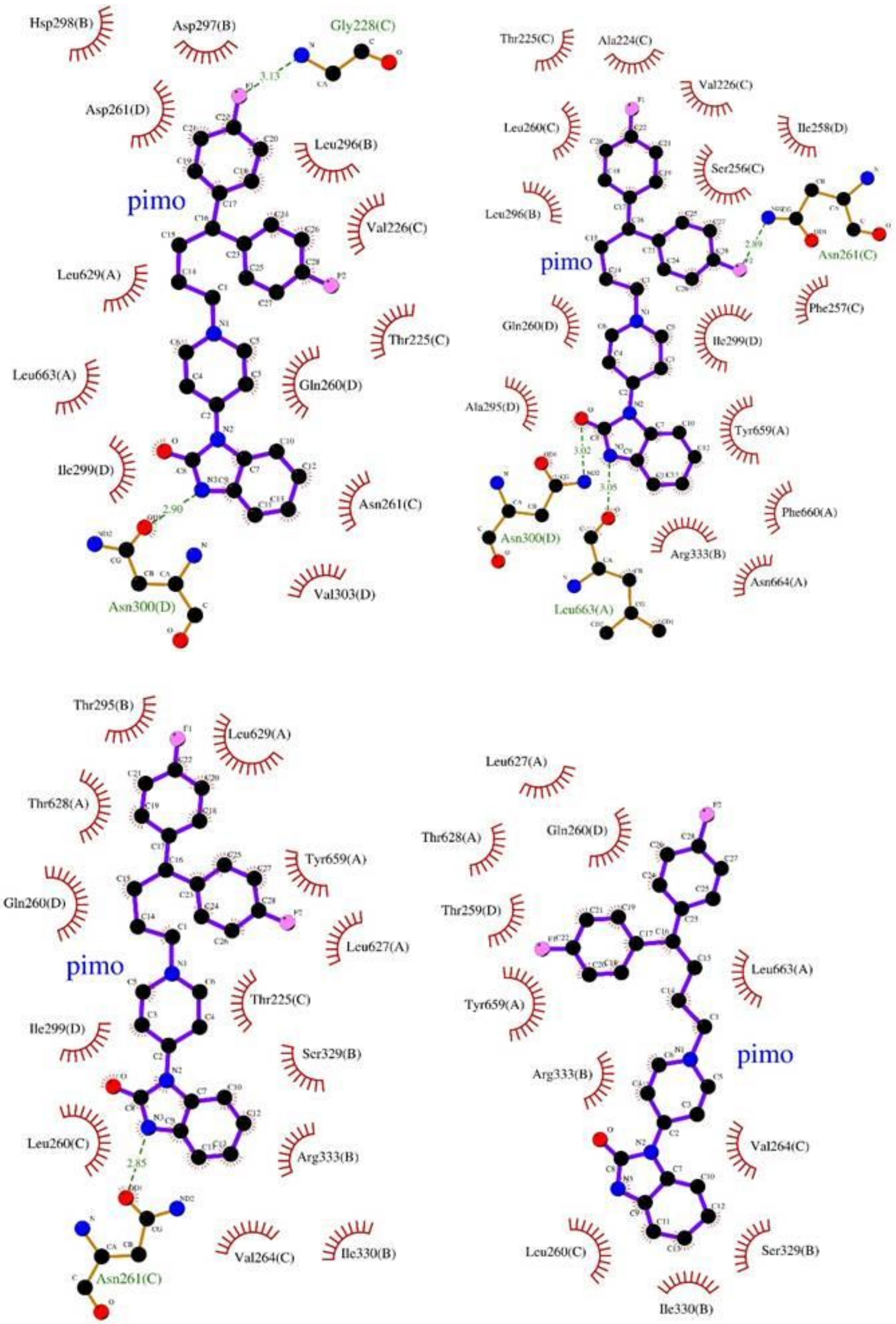

Figure S2 Binding mode of the four replicas. Radial spikes indicate hydrophobic interactions, while dotted lines indicate hydrogen bonds.
